# Asymmetries of Force and Power During Single-Leg Counter Movement Jump in Young Adult Females and Males

**DOI:** 10.3390/s25164995

**Published:** 2025-08-12

**Authors:** Jarosław Kabaciński, Joanna Gorwa, Waldemar Krakowiak, Michał Murawa

**Affiliations:** Department of Biomechanics, Poznan University of Physical Education, 61-871 Poznan, Poland

**Keywords:** asymmetry, force, power, single-leg counter movement jump, gender

## Abstract

Background/Objectives: Inter-limb asymmetry of a given variable for vertical jumps is commonly assessed in both healthy individuals and those undergoing rehabilitation post-injury. The aim of this study was to compare the asymmetry index between the take-off and landing of a single-leg counter movement jump (CMJ), as well as between females and males. Methods: Twenty-three healthy females (age: 21.5 ± 1.6 years) and twenty-three healthy males (age: 21.1 ± 1.8 years) participated in this study. The assessment of two asymmetry indices (AI1 and AI2) was conducted for the peak vertical ground reaction force (PVGRF) and maximum power (MP) during single-leg CMJ take-offs and landings performed on the force platform. Results: The analysis showed significant main effects (*p* < 0.001) for the phase factor (only AI2) and for the gender factor (only AI1). Moreover, there was a non-significant interaction effect between the phase factor and gender factor (*p* = 0.476). Pairwise comparisons revealed significant differences in the values of (1) AI2 between the take-off and landing (*p* < 0.001) and (2) AI1 between females and males (*p* < 0.001). Conclusions: Findings showed significant effects of the phase factor (only for AI2) and gender factor (only for AI1) on the magnitude of inter-limb asymmetry during single-leg CMJs. Furthermore, this study reported the significantly higher asymmetry of the PVGRF and MP for landing than take-off, which may result from difficulties in controlling the jumper’s landing technique on one foot at higher velocity. In addition, the assessment of asymmetry for single-leg CMJs using AI1 should be performed separately for females and males, as opposed to AI2. Participants of both genders generally demonstrated a higher AI level for the power than for the force.

## 1. Introduction

Many biomechanical tests often involve comparing the function of one limb with the contralateral limb to determine the magnitude of asymmetry. The percentage difference of a given variable between limbs is provided by the so-called asymmetry index (AI), defined using various formulas. Among the various indices, the calculations provide the standard ratio index [1,2], the symmetry index [3,4,5,6,7], the strength asymmetry index [8,9], the Vagenas and Hoshizaki formula [10], and the formula containing the natural logarithm [11].

The AI value above 10% or 15% indicates a significant inter-limb asymmetry that may contribute to reduced performance of the weaker limb and increased risk of injury [2,12,13,14]. Researchers linked an increased asymmetry to factors such as overuse of the dominant limb in specific sports [15,16], previous injuries to one limb [17,18,19], the intensity of plyometric exercise involving jumps [20,21], and gender [22,23,24]. In some sports (e.g., football), above-threshold asymmetry of strength or power may impair exercise performance and negatively affect athletic outcomes [16,25,26,27,28]. In turn, in the athlete after anterior cruciate ligament (ACL) reconstruction, excessive asymmetry may hinder a timely return to training and increase the risk of re-injury [13,18,19,29].

Vertical jumps such as the counter movement jump (CMJ), squat jump (SJ), and drop jump (DJ) are commonly performed using a double-leg technique. However, the level of force and power can also be assessed during single-leg take-offs and landings of CMJs, SJs, or DJs. The evaluation of both variables for each lower extremity separately in these jumps is crucial, as many sport-specific movement patterns are unilateral in nature [30]. Therefore, single-leg vertical jumps have gained wide utility for examining inter-limb asymmetry in both healthy athletes and athletes rehabilitated after ACL reconstruction [13,30,31,32].

Previous studies have focused on assessing the asymmetry of selected variables during vertical jumps performed on a force platform in healthy individuals [13,21,22,25,26,30,33,34,35,36] and athletes with ACL injury [17,19,31,37,38]. Considering the take-off and landing, Paterno et al. [31] investigated the asymmetry of the peak vertical ground reaction force (PVGRF) during DJs in female athletes after ACL reconstruction. Furthermore, Cone et al. [21] determined the differences in the asymmetry index for PVGRF between the take-off and landing phases of the double-leg CMJs and double-leg DJs in men. However, the analysis of the available papers indicates incomplete data on the influence of concentric contraction (take-off) or eccentric contraction (landing) and gender on the magnitude of inter-limb asymmetry of a given variable in single-leg CMJs. Therefore, the aim of the study was to compare the asymmetry index between the take-off and landing, as well as between females and males, for the force and power developed during the single-leg CMJ.

## 2. Materials and Methods

### 2.1. Participants

A total of forty-six students from Poznan University of Physical Education participated in this study. This group included twenty-three females (age: 21.5 ± 1.6 years, body mass: 59.0 ± 5.0 kg, and body height: 1.66 ± 0.05 m) and twenty-three males (age: 21.1 ± 1.8 years, body mass: 72.5 ± 9.2 kg, and body height: 1.79 ± 0.07 m). The sample size was calculated using G*Power software (version 3.1.9.7). For an effect size of 0.5, a power of 0.80, and an alpha level of 0.05, a total sample size of 34 was obtained.

All participants were healthy and recreationally active. The International Physical Activity Questionnaire (IPAQ) was used to determine the physical activity of the participants [39]. The IPAQ results indicated that the activity level was high for all participants and sufficient in 6 participants. In addition, each student declared the right lower extremity (LE) as the functionally dominant during various movements (e.g., jumping, kicking). This dominance was confirmed in all participants by a ball-kicking test performed in the laboratory. Hence, the dominant LE (D) and the non-dominant LE (ND) were adopted. The inclusion criteria included (1) age between 18 and 24 years, (2) non-professional sports practice, (3) no history of ankle, knee, hip, or back injuries (one year before testing), (4) lack of potential medical problems, and (5) at least a sufficient level of physical activity based on the IPAQ. All participants were familiarized with the experimental procedures and provided informed consent to participate in this study. The study was conducted in accordance with the Declaration of Helsinki, and the protocol was approved by the Ethics Committee of the Poznan University of Medical Sciences (number 546/16, 10 June 2022).

### 2.2. Data Collection

The stationary force platform 800 Hz (BP400600, AMTI, Watertown, MA, USA) was used. The ground reaction forces were collected using the BTS Smart Capture software (version Smart-D, BTS Bioengineering, Milan, Italy). The single-leg counter movement jump (CMJ) was used to compare the peak vertical ground reaction force (PVGRF) and maximum power (MP) between the dominant LE (D) and non-dominant LE (ND) for both the take-off and landing. These tests were conducted over four days from 10 a.m. to 2 p.m. (June 2023). Measurements were preceded by a 10 min warm-up involving treadmill running as well as static and dynamic stretching exercises. Each student was instructed to keep their hands on their hips, jump as high as possible, take off from one foot, and land on the same foot during the CMJ. After a few trials, the jumper performed six successful single-leg CMJs, i.e., three for D and three for ND, alternately (first one LE and then the opposite LE) and randomly (first D or ND). The participant started the jump from an upright position (standing phase) and flexed their knee joint to an angle of approximately 90° (braking phase). Half-minute rest periods between these jumps and approximately one-minute rest periods between the CMJ trials and successful CMJs were assumed.

### 2.3. Data Analysis

The VGRF data vs. time for the 276 trials were exported as files with the .xls extension. Then, in Microsoft Excel 2019, the velocity values were calculated based on numerical integration according to Simpson’s method. The following formulas were used:

(1) $$a_{z}=\frac{R_{z}}{m}-g$$

where m represents body mass, R_z_ is the vertical ground reaction force, and g is the gravity acceleration (9.80665 m·s^−2^),

(2) $$v_{z}(t)=v_{oz}+\int a_{z}(t)dt$$

where v_z_(t) represents vertical velocity vs. time, v_oz_ is the initial vertical velocity, and a_z_(t) is vertical acceleration vs. time,

(3) $$P_{z}(t)=R_{z}(t)\cdot v_{z}(t)$$

where P_z_(t) represents vertical mechanical power vs. time.

In this study, the asymmetry index (AI) values were calculated using Formula (4) [17,40] and Formula (5) [8,9,10].

(4) $$AI1=\frac{X_{D}-X_{ND}}{\max(X_{D},X_{ND})}\cdot 100\%$$

(5) $$AI2=\frac{\left|X_{D}-X_{ND}\right|}{\max(X_{D},X_{ND})}\cdot 100\%$$

where X_D_ and X_ND_ are the values of a given variable for the D and ND, respectively, related to a single trial.

### 2.4. Statistical Analysis

Statistical analysis was conducted using the IBM SPSS Statistics software for Windows, version 30.0 (Armonk, NY, USA: IBM Corp). The Shapiro–Wilk test was used to verify the distribution of variables. The paired samples *t*-test for the comparisons between the LEs was used. The mixed-factorial ANOVA with two factors (phase [take-off or landing] × gender [female or male]) for the PVGRF and MP was performed. Sphericity was examined using the Mauchly test. A Bonferroni correction for multiple pairwise comparisons was used. The effect size for the ANOVA test was determined using the partial eta-squared (η^2^). According to the Cohen guidelines, values of η^2^ were small for 0.01, medium for 0.06, and large for 0.14 [41]. Significance level alpha was set at *p* < 0.05.

## 3. Results

The means and standard deviations of the variables in females and males are presented in Table 1.

Comparisons between LEs revealed significant differences in the values of PVGRF (*p* = 0.001) and MP (*p* = 0.01) for take-off in females and non-significant differences in the values of (1) PVGRF and MP for landing in females (*p* > 0.05) and (2) PVGRF and MP for take-off and landing in males (*p* > 0.05).

Effect sizes and *p*-values of the mixed-factorial ANOVA for the three factors and interactions are presented in Table 2 and Table 3.

Considering the analyzed variables, the sphericity (W = 1.0; *p* = 1.000) of all factors and all interactions was fulfilled.

Analysis showed the non-significant main within-subject effect (*p* > 0.05) for the AI1 and the significant main within-subject effect (*p* < 0.05) for the AI2 at F_1,44_ = 22.3 (PVGRF) and F_1,44_ = 25.5 (MP). The significant between-subject main effect (*p* < 0.05) at F_1,44_ = 9.3 for the AI1 (MP) only was observed. A non-significant interaction effect (*p* > 0.05) for both variables was found.

Results of the η^2^ indicated a (1) large effect size for phase factor (AI1 for MP and AI2 for PVGRF and MP), gender factor (AI1 for PVGRF and MP), and interaction factor (AI1 and AI2 for MP); (2) medium effect size for gender factor (AI2 for PVGRF) and interaction factor (AI1 for PVGRF); and (3) small effect size for phase factor (AI1 for PVGRF), gender factor (AI2 for MP), and interaction factor (AI2 for PVGRF).

**Figure 1:**
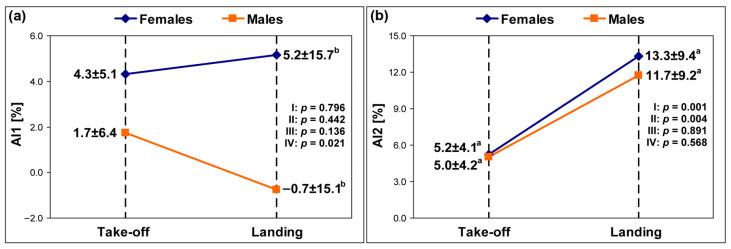
The means and standard deviations of (**a**) the AI1 and (**b**) the AI2 for the PVGRF. AI1—the first asymmetry index, AI2—the second asymmetry index, PVGRF—the peak vertical ground reaction force, I—take-off vs. landing for females, II—take-off vs. landing for males, III—females vs. males for take-off, IV—females vs. males for landing, a—significance for comparisons between take-off and landing, b—significance for comparisons between females and males.

**Figure 2:**
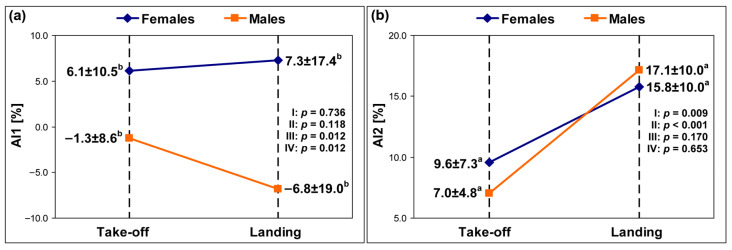
The means and standard deviations of (**a**) the AI1 and (**b**) the AI2 for the MP. AI1—the first asymmetry index, AI2—the second asymmetry index, MP—the maximum power, I—take-off vs. landing for females, II—take-off vs. landing for males, III—females vs. males for take-off, IV—females vs. males for landing, a—significance for comparisons between take-off and landing, b—significance for comparisons between females and males.

The means and standard deviations of the AI1 and AI2 in females and males are presented in Figure 1 and Figure 2. The analysis revealed significantly higher values of (1) AI1 in females than males for the take-off phase by 7.4% (MP) as well as the landing phase by 5.9% (PVGRF) and by 14.1% (MP) and (2) AI2 for the landing phase than the take-off phase by 8.1% (PVGRF) and by 6.2% (MP) in females as well as by 6.7% (PVGRF) and by 10.1% (MP) in males.

The pairwise comparisons also showed non-significant differences in the AI1 values: (1) between the take-off and landing phases in females and males for both variables (*p* > 0.05) and (2) between females and males during the take-off phase for PVGRF only (*p* > 0.05). Considering the AI2 values, non-significant differences between females and males for both variables were found (*p* > 0.05).

## 4. Discussion

This study compared two asymmetry indices for force (PVGRF) and power (MP) between the take-off and landing phases in single-leg CMJs, as well as between female and male students. The asymmetry index AI1—calculated according to Formula (4) [17,40], i.e., the ratio of D-ND and the greater value of D and ND—and the asymmetry index AI2—calculated according to Formula (5) [8,9,10], i.e., the ratio of |D-ND| and the greater value of D and ND—were used for the analysis.

The analysis showed generally non-significant differences in the mean results of both variables between D and ND, indicating similar force and power of one LE to the contralateral LE. In particular, the AI1 results indicated a non-significant asymmetry for PVGRF and MP (below the normative threshold of 10%) in participants, both for the take-off phase (mean AI1 in range: −1.3–6.1%) and landing phase (mean AI1 in range: −6.8–7.3%). Thus, expected AI values were obtained because the evaluation included healthy, recreationally active women and men. Most female students demonstrated slightly greater force and power during take-offs and landings for D than for ND (except for PVGRF for take-offs). In turn, the majority of male students performed non-significantly stronger and more powerful take-offs from ND and landings on ND compared to D, which resulted in a negative mean AI1 value. In contrast, the AI2 only provides positive results due to the absolute value in the numerator of Formula (5). Therefore, averaging negative and positive AI1 values led to a greater data dispersion (larger standard deviation) compared to AI2.

The findings revealed a significant main effect for the factor phase and thus its influence on the level of AI in both groups. However, only AI2 results indicated significantly greater PVGRF and MP asymmetry for landings than for take-offs in both females and males. This trend was not observed for AI1, as the mean values of this index for landings were considerably lower than those of AI2. In contrast to AI1, the AI2 formula contains an absolute value in the numerator; hence, it always provides a result greater than zero. Undoubtedly, the use of different AIs influenced the divergence of their results for comparisons between the take-off and landing phases.

The increased asymmetry in force and power observed for the landing phase of the single-leg CMJ can be attributed, among others, to poorer stability of the ND joints during the high eccentric loads. Greater dynamic loads imposed on the LE in the landing phase result from large VGRF and increased movement velocity. Additionally, a valgus knee alignment of the jumper in the frontal plane often occurs [42]. These factors contribute to greater instability of the knee joint, which leads to difficulties in controlling landing mechanics. Therefore, this study also assessed AI for power, i.e., a variable defined as the product of VGRF and velocity. In both females and males, higher AI values were found for power than for force due to the influence of movement velocity.

Some authors have identified knee instability during landings as a risk factor for non-contact ACL injuries, particularly in female athletes [21,24,43,44]. The ACL in women absorbs greater external loads due to the more extended knee position during the ground contact [45]. Therefore, a crucial strategy for preventing ACL injuries is the correct landing technique, minimizing unfavorable loads on the knee joint structures [43,46,47,48]. Furthermore, athletes should also perform soft and stable landings during eccentric exercises, such as CMJs and DJs, with both legs and one leg. In addition, such plyometric training, used to improve muscle capacity, knee stability, and neuromuscular control during the landing, is considered an important part of the rehabilitation program of athletes after ACL reconstruction [20,21,29,49].

Previous studies have assessed inter-limb force asymmetry during take-off and landing in vertical jumps on the force platform [21,26,31]. Furthermore, Cone et al. [21] compared AI between take-off and landing phases, and similarly to the present study, they reported significantly greater AI values for PVGRF in the landing phase than in the take-off phase of the CMJ. Surprisingly, Cone et al. [21] obtained such results using AI1 during bilateral jumps performed only by men on two force platforms. However, athletes such as volleyball, basketball, and soccer players frequently take off from one LE and land on one LE during games. There are also situations when a jumping athlete asymmetrically loads the LE, coming into contact with the ground with one foot before the other foot [21,31].

The importance of using AI2 is emphasized by the divergent results between both indices for comparisons between female students and male students. The analysis showed the influence of gender on the results of AI1 and the lack of such a tendency for AI2. In females, a non-significantly higher mean value of AI1 was observed than in males for both take-offs and landings. This difference may be attributed to the different movement biomechanics of women during the CMJ, determined by their different body structure in comparison with men. Considering volleyball jumps, Salci et al. [46] observed gender differences in the values of dynamic, kinetic, and kinematic variables for the knee, hip, and ankle during landings. However, AI2 results indicated non-significant differences in values of this index between both groups. Thus, the selection of the type of AI plays a role in determining the level of asymmetry in participants differentiated by gender. When using AI1, researchers should assess force and power asymmetries in vertical jumps separately for women and men. In addition, comparisons revealed a non-significant interaction effect between the gender factor and phase factor for AI1 and AI2; thus, the difference in AI values between the landing and take-off is not dependent on gender. In both females and males, greater asymmetries in force and power were generally observed for the landing phase than for the take-off phase.

This study had limitations. First, the refusals of some participants due to injuries, illness, and travel prevented the tests from being conducted in the second session with the same group size as in the first session. Second, the study included only recreationally active students, thus excluding athletes. Therefore, these findings are difficult to relate to the data obtained by the athletes.

## 5. Conclusions

A review of available papers suggests that this study is the first to compare different asymmetry indices of the force and power between the take-off and landing phases, as well as between females and males during the single-leg CMJ. For both groups, the findings revealed a significantly greater asymmetry of the PVGRF and MP for the landing than for the take-off, only in the case of AI2, consistent with the Vagenas and Hoshizaki formula [10]. It was explained that this trend, among others, may result from difficulties in controlling the jumper’s landing technique on one foot at higher velocity compared to the take-off. Furthermore, a higher level of power asymmetry than force asymmetry was observed both in females and males due to the influence of velocity. In addition, it was proposed that the assessment of inter-limb asymmetry using the AI1 for the single-leg CMJ should be conducted separately in women and men, as opposed to AI2. Further tests involving populations of healthy individuals and athletes after ACL reconstruction, performing various vertical jumps on a force platform, are needed.

## Figures and Tables

**Table 1:** The means and standard deviations of PVGRF and MP during the take-off and landing phases.

Variable	Phase	Females		Males	
		Non-Dominant	Dominant	Non-Dominant	Dominant
PVGRF (N)	Take-off	1163.9 ± 129.8	1217.8 ± 134.9	1572.4 ± 161.6	1597.0 ± 112.8
	Landing	2033.7 ± 432.1	2149.5 ± 367.8	2737.4 ± 463.1	2705.7 ± 401.8
MP (W)	Take-off	1733.0 ± 223.2	1853.1 ± 236.6	2580.3 ± 290.6	2548.7 ± 290.3
	Landing	2948.0 ± 514.0	3206.3 ± 502.3	4311.3 ± 711.6	4053.8 ± 1016.6

Notes: PVGRF—the peak vertical ground reaction force, MP—the maximum power.

**Table 2:** Effect sizes of the mixed-factorial ANOVA for the AI1.

Factor	PVGRF		MP	
	η^2^	*p*	η^2^	*p*
Phase	0.003	0.717	0.018	0.379
Gender	0.058	0.108	0.175	0.004 *
Phase × Gender	0.012	0.467	0.041	0.178

Notes: PVGRF—the peak vertical ground reaction force, MP—the maximum power, AI1—the first asymmetry index, η^2^—the partial eta-squared, *—significant difference.

**Table 3:** Effect sizes of the mixed-factorial ANOVA for the AI2.

Factor	PVGRF		MP	
	η^2^	*p*	η^2^	*p*
Phase	0.336	<0.001 *	0.367	<0.001 *
Gender	0.008	0.547	0.002	0.743
Phase × Gender	0.005	0.656	0.032	0.236

Notes: PVGRF—the peak vertical ground reaction force, MP—the maximum power, AI2—the second asymmetry index, η^2^—the partial eta-squared, *—significant difference.

## Data Availability

The data are available upon request from the corresponding author.

## References

[1] Ganguli S., Mukherji P., Bose K.S. (1974). Gait evaluation of unilateral below-knee amputees fitted with patellar-tendon-bearing prostheses. J. Indian Med. Assoc..

[2] Schiltz M., Lehance C., Maquet D., Bury T., Crielaard J.M., Croisier J.L. (2009). Explosive strength imbalances in professional basketball players. J. Athl. Train..

[3] Robinson R.O., Herzog W., Nigg B.M. (1987). Use of force platform variables to quantify the effects of chiropractic manipulation on gait symmetry. J. Manip. Physiol. Ther..

[4] Wong P.L., Chamari K., Chaouachi A., Mao D.W., Wisløff U., Hong Y. (2007). Difference in plantar pressure between the preferred and non-preferred feet in four soccer-related movements. Br. J. Sports Med..

[5] Bell D.R., Sanfilippo J.L., Binkley N., Heiderscheit B.C. (2014). Lean mass asymmetry influences force and power asymmetry during jumping in collegiate athletes. J. Strength Cond. Res..

[6] Sugiyama T., Kameda M., Kageyama M., Kiba K., Kanehisa H., Maeda A. (2014). Asymmetry between the dominant and non-dominant legs in the kinematics of the lower extremities during a running single leg jump in collegiate basketball players. J. Sports Sci. Med..

[7] Marshall B., Franklyn-Miller A., Moran K., King E., Richter C., Gore S., Strike S., Falvey É. (2015). Biomechanical symmetry in elite rugby union players during dynamic tasks: An investigation using discrete and continuous data analysis techniques. BMC Sports Sci. Med. Rehabil..

[8] Bishop C., Read P., Lake J., Chavda S., Turner A. (2018). Interlimb asymmetries: Understanding how to calculate differences from bilateral and unilateral tests. Strength Cond. J..

[9] Impellizzeri F.M., Rampinini E., Maffiuletti N., Marcora S.M. (2007). A vertical jump force test for assessing bilateral strength asymmetry in athletes. Med. Sci. Sports Exerc..

[10] Vagenas G., Hoshizaki B. (1992). A multivariable analysis of lower-extremity kinematic asymmetry in running. Int. J. Sport Biomech..

[11] Plotnik M., Giladi N., Balash Y., Peretz C., Hausdorff J.M. (2005). Is freezing of gait in Parkinson’s disease related to asymmetric motor function?. Ann. Neurol..

[12] Brumitt J., Heiderscheit B.C., Manske R.C., Niemuth P.E., Rauh M.J. (2013). Lower extremity functional tests and risk of injury in division III collegiate athletes. Int. J. Sports Phys. Ther..

[13] Vaisman A., Guiloff R., Rojas J., Delgado I., Figueroa D., Calvo R. (2017). Lower limb symmetry: Comparison of muscular power between dominant and nondominant legs in healthy young adults associated with single-leg-dominant sports. Orthop. J. Sports Med..

[14] Helme M., Tee J., Emmonds S., Low C. (2021). Does lower-limb asymmetry increase injury risk in sport? A systematic review. Phys. Ther. Sport..

[15] Markou S., Vagenas G. (2006). Multivariate isokinetic asymmetry of the knee and shoulder in elite volleyball players. Eur. J. Sport Sci..

[16] Michailidis Y., Stafylidis A., Mandroukas A., Kyranoudis A.E., Antoniou G., Kollias R., Kanaras V., Bamplekis C., Vardakis L., Semaltianou E. (2025). Correlation of the asymmetry index from the single-leg countermovement jump with the asymmetry index from isokinetic strength in elite youth football players. Appl. Sci..

[17] Jordan M.J., Aagaard P., Herzog W. (2015). Lower limb asymmetry in mechanical muscle function: A comparison between ski racers with and without ACL reconstruction. Scand. J. Med. Sci. Sports..

[18] Wiggins A.J., Grandhi R.K., Schneider D.K., Stanfield D., Webster K.E., Myer G.D. (2016). Risk of secondary injury in younger athletes after anterior cruciate ligament reconstruction: A systematic review and meta-analysis. Am. J. Sports Med..

[19] Chen P., Wang L., Dong S., Ding Y., Jia S., Zheng C. (2024). Can symmetry of single-leg vertical jump height represent normal lower limb biomechanics of athletes after anterior cruciate ligament reconstruction?. Sports Health.

[20] Chmielewski T.L., George S.Z., Tillman S.M., Moser M.W., Lentz T.A., Indelicato P.A., Trumble T.N., Shuster J.J., Cicuttini F.M., Leeuwenburgh C. (2016). Low- versus high-intensity plyometric exercise during rehabilitation after anterior cruciate ligament reconstruction. Am. J. Sports Med..

[21] Cone S.M., Lee S. (2021). Lower limb force asymmetries during landing and jumping exercises: A pilot study. Int. J. Exerc. Sci..

[22] Stephens T.M., Lawson B.R., Reiser R.F. (2005). Bilateral asymmetries in max effort single-leg vertical jumps. Biomed. Sci. Instrum..

[23] Stephens T.M., Lawson B.R., DeVoe D.E., Reiser R.F. (2007). Gender and bilateral differences in single-leg countermovement jump performance with comparison to a double-leg jump. J. Appl. Biomech..

[24] Hewett T.E., Ford K.R., Hoogenboom B.J., Myer G.D. (2010). Understanding and preventing acl injuries: Current biomechanical and epidemiologic considerations—Update 2010. N. Am. J. Sports Phys. Ther..

[25] Bishop C., Coratella G., Beato M. (2021). Intra- and inter-limb strength asymmetry in soccer: A comparison of professional and under-18 players. Sports.

[26] Bishop C., Read P., McCubbine J., Turner A. (2021). Vertical and horizontal asymmetries are related to slower sprinting and jump performance in elite youth female soccer players. J. Strength Cond. Res..

[27] Lin J., Shen J., Zhou A., Badicu G., Grosz W.R. (2022). The effects of inter-limb asymmetry on change of direction performance: A systematic review. Symmetry.

[28] Michailidis Y. (2023). Relation of jump and change of direction inter-limb asymmetries with fitness in youth male soccer players. Medicina.

[29] Buckthorpe M., Della Villa F. (2021). Recommendations for plyometric training after ACL reconstruction—A clinical commentary. Int. J. Sports Phys. Ther..

[30] Hewit J.K., Cronin J.B., Hume P.A. (2012). Asymmetry in multi-directional jumping tasks. Phys. Ther. Sport..

[31] Paterno M.V., Ford K.R., Myer G.D., Heyl R., Hewett T.E. (2007). Limb asymmetries in landing and jumping 2 years following anterior cruciate ligament reconstruction. Clin. J. Sport Med..

[32] Ebben W.P., Flanagan E., Jensen R.L. (2009). Bilateral facilitation and laterality during the countermovement jump. Percept. Mot. Skills.

[33] McElveen M.T., Riemann B.L., Davies G.J. (2010). Bilateral comparison of propulsion mechanics during single-leg vertical jumping. J. Strength Cond. Res..

[34] Kobayashi Y., Kubo J., Matsubayashi T., Matsuo A., Kobayashi K., Ishii N. (2013). Relationship between bilateral differences in single-leg jumps and asymmetry in isokinetic knee strength. J. Appl. Biomech..

[35] Bishop C., de Keijzer K.L., Turner A.N., Beato M. (2023). Measuring interlimb asymmetry for strength and power: A brief review of assessment methods, data analysis, current evidence, and practical recommendations. J. Strength Cond. Res..

[36] Wang P., Qin Z., Zhang M. (2025). Association between pre-season lower limb interlimb asymmetry and non-contact lower limb injuries in elite male volleyball players. Sci. Rep..

[37] Taylor J.B., Westbrook A.E., Head P.L., Glover K.M., Paquette M.R., Ford K.R. (2020). The single-leg vertical hop provides unique asymmetry information in individuals after anterior cruciate ligament reconstruction. Clin. Biomech..

[38] Ciccodicola E.M., Hanson A.M., Roberts S.E., Katzel M.J., Wren T.A.L. (2025). Biomechanics and performance of single-leg vertical and horizontal hop in adolescents post-anterior cruciate ligament reconstruction. Biomechanics.

[39] Craig C.L., Marshall A.L., Sjöström M., Bauman A.E., Booth M.L., Ainsworth B.E., Pratt M., Ekelund U., Yngve A., Sallis J.F. (2003). International physical activity questionnaire: 12-country reliability and validity. Med. Sci. Sports Exerc..

[40] Cheung R.T., Smith A.W., Wong D.P. (2012). H:Q ratios and bilateral leg strength in college field and court sports players. J. Hum. Kinet..

[41] Cohen J. (1988). Statistical Power Analysis for the Behavioral Sciences.

[42] Paterno M.V., Schmitt L.C., Ford K.R., Rauh M.J., Myer G.D., Huang B., Hewett T.E. (2010). Biomechanical measures during landing and postural stability predict second anterior cruciate ligament injury after anterior cruciate ligament reconstruction and return to sport. Am. J. Sport Med..

[43] Griffin L.Y., Agel J., Albohm M.J., Arendt E.A., Dick R.W., Garrett W.E., Garrick J.G., Hewett T.E., Huston L., Ireland M.L. (2000). Noncontact anterior cruciate ligament injuries: Risk factors and prevention strategies. J. Am. Acad. Orthop. Surg..

[44] Myer G.D., Brent J.L., Ford K.R., Hewett T.E. (2011). Real-time assessment and neuromuscular training feedback techniques to prevent ACL injury in female athletes. Strength Cond. J..

[45] Colby S., Francisco A., Yu B., Kirkendall D., Finch M., Garrett W. (2000). Electromyographic and kinematic analysis of cutting maneuvers. Implications for anterior cruciate ligament injury. Am. J. Sports Med..

[46] Salci Y., Kentel B.B., Heycan C., Akin S., Korkusuz F. (2004). Comparison of landing maneuvers between male and female college volleyball players. Clin. Biomech..

[47] Reeser J.C., Verhagen E., Briner W.W., Askeland T.I., Bahr R. (2006). Strategies for the prevention of volleyball related injuries. Br. J. Sports Med..

[48] Blackburn J.T., Padua D.A. (2009). Sagittal-plane trunk position, landing forces, and quadriceps electromyographic activity. J. Athl. Train..

[49] Kasmi S., Zouhal H., Hammami R., Clark C.C.T., Hackney A.C., Hammami A., Chtara M., Chortane S.G., Salah F.Z.B., Granacher U. (2021). The effects of eccentric and plyometric training programs and their combination on stability and the functional performance in the post-ACL-surgical rehabilitation eriod of elite female athletes. Front. Physiol..

